# Palliative Care in the Health System of Iran: A Review of the Present Status and the Future Challenges

**DOI:** 10.31557/APJCP.2020.21.3.845

**Published:** 2020-03

**Authors:** Salman Barasteh, Maryam Rassouli, Akram Parandeh, Amir Vahedian-Azimi, Rohallah Zaboli, Morteza Khaghanizadeh

**Affiliations:** 1 *Medicine Quran and Hadith Research Center, *; 3 *Trauma Research Center, Faculty of Nursing, *; 4 *Health Management Research Center, Baqiyatallah University of Medical Sciences, *; 2 *Cancer Research Center, Shahid Beheshti University of Medical Sciences, Tehran, Iran.*

**Keywords:** Palliative care, challenge- health system of Iran- non-communicable disease (NCD)- chronic disease

## Abstract

**Objectives:**

In the near future, the health system of Iran will face serious public health challenges means increase in the elderly population and the rate of chronic diseases. Therefore, it is anticipated that providing palliative care for chronic diseases will be one of the priorities of the country’s health system. The purpose of the present study was to explain the present status and the future challenges of providing palliative care in the health system of Iran and help policy-makers to create a future roadmap by presenting a picture of the present status.

**Methods:**

In this qualitative study, 17 semi-structured interviews were conducted with policy-makers, researchers, and managers of the centers providing palliative care in 2018-2019. Interviews were analyzed using directed content analysis based on the Public Health Strategy and framework analysis.

**Results:**

According to the WHO Public Health Strategy, palliative care challenges categorized in 13 subcategory and four main category include policy-making, program implementation, comprehensive education and drug availability.

**Conclusion:**

Providing palliative care that is currently dispersed in some centers does not meet the needs of chronic diseases. Establishing the palliative care system as one of the major goals of the health system of Iran is possible through reforming the fourfold structure of policy-making, implementation, education and drug availability. Therefore, it is suggested that authorities perform comprehensive and systematic management of challenges using foresight methods.

## Introduction

Iran, with 82 million populations (Aloosh et al., 2019), is the 18^th^ country in the world, the second country in the Middle East and upon to the World Bank classification is among the upper middle-in-come countries (Hassanipour et al., 2018). According to the latest report of the World Health Organization (WHO) in 2016, life expectancy is 75.5 years (Mojen et al., 2018). Based on Bloomberg’s ranking, Iran has been ranked as the 45^th^ country in the world in terms of effective health system. The Ministry of Health and Medical Education (MOHME) is responsible for the health stewardship and centralized sovereignty. The activities of this ministry have shown improved health status over the past few decades (Rafiei et al., 2015). Nevertheless, the health system of Iran faces major challenges. The major challenges of the health system of Iran include increasing the elderly population, increasing non-communicable diseases (NCDs), changing lifestyle, as well as increasing health costs, maintaining and increasing quality of care, and patients’ place of death and dignity (Jabbari et al., 2019). In 2016, NCDs have killed 287,000 people in Iran. The 2016 census represented 11 percent of Iran’s population over 60 years; it is projected that by 2050, a quarter of Iran’s population will be over 60 years old (WHO, 2019). 7% of gross domestic product (GDP) is allocated to the health sector (Mahboub-Ahari et al., 2019), and total expenditure on health per capita in 2014 was $ 1,082 (WHO, 2019). However, GDP growth and public health in Iran are influenced by many factors; for example, the November 5, 2018 US sanctions dramatically affect the health of individuals at the individual and social levels through social determinants of health (SDH) and drug availability (Aloosh et al., 2019). In these circumstances, it is important to develop solutions to overcome the challenges raised.

*Present status of palliative care in Iran*

The pivotal point in addressing these challenges is palliative care(PC) based on three levels of prevention and consistency with the definition of Universal Health Coverage (UHC), i.e. access to key health interventions that include promotion, prevention, treatment, rehabilitation and PC (Koohpayehzadeh and Kassaeian, 2018). PC as one of the dimensions of the UHC is an approach that improves the quality of life of patients and their families in the face of life-threatening disease problems by preventing and alleviating pain based on initial identification, assessment and treatment of pain and other physical, mental, social and spiritual problems. Regarding the high prevalence of chronic diseases, the increasing number of patients requiring PC, and the development of services, the necessity of PC is justified that makes patients requiring PC identify and benefit from primary care (Quill and Abernethy, 2013). There have been several studies on PC challenges in Iran, each with a different approach to PC challenges in Iran. In the study by Khoshnazar et al., (2019), two challenges of lack of PC definition in the health system and weaknesses of PC providers (Khoshnazar et al., 2016) are presented as the most important PC challenges. In their study, Ansari et al., (2019) stated that the process challenges of providing PC including weaknesses in policy-making, standard care provider, and the use of educational and research approaches are the main PC challenges. However, Jabbari et al., (2019) showed that the challenges, opportunities, and weaknesses of PC in the country is based on the context of the country and has been explained according to the current and possible conditions of the country. Reviewing the literature, it can be concluded that despite several studies, none of them have been conducted according to the possible future conditions, although such a suggestion has been made by Rassouli and Sajjadi, (2016a), no response has been provided. A review of studies suggests that PC challenges in the future of the health system of Iran have not yet been well elucidated. Therefore, given the fundamental emerging challenges of aging, and exposure to NCDs in the not too distant future, addressing the above-mentioned challenges will have a serious impact on the formation of future health care patterns and demands; therefore, some studies are required to deal with this phenomenon. So in order to integrate the PC effectively into the community, the four dimensions of the WHO Public Health Strategy model, including appropriate policies, adequate availability of drugs, training of community and health services staff, and implementation of PC services at all levels of society should be taken into account (Stjernswärd et al., 2007). This study aimed to explain the present status and the future challenges of PC delivery in the health system of Iran based on WHO Public Health Strategy (PHS).

## Materials and Methods

*Design*

The present qualitative study was conducted from December 2018 to May 2019 based on directed content analysis using the framework analysis (Ritchie and Spencer, 2002). 

*Participants*

17 policy-makers, decision-makers, faculty members, researchers, centre manager and PC providers such as physician and other fields were selected by purposeful sampling. The main criterion for selecting the participants was having sufficient experience and expertise in PC and consent to participate in the study process. After obtaining consent and providing coordination with the participants, the interview was conducted. Interviews ended after data saturation. After completing the sampling, two further interviews were conducted, and it was ensured that no further findings were added during the content analysis process. Interviews were conducted in the places where the participants tended to, with prior coordination, or at their workplace.

*Data collection*

Semi-structured interviews were conducted by the first author. The interview time was 17 to 60 minutes. The interview process was based on four stages: (1) Orientation phase: In this phase, the researcher introduced himself / herself, title, goals, and the probable time of interview, and recording permission and permission to refer back to the participant were obtained from the participants, (2) Main question phase: In this phase, the main research question (How do you see the present and the future challenges of PC in the health system of Iran?) was asked from the participant, (3) Probing phase: In this phase, given the participant’s stated experiences, the following questions raised by the participant’s stated experiences were asked. Examples of these questions were as follows: Since the concept of PC was introduced in the country until now, what changes have you seen? What are the reasons for this progress? What needs to happen if the PC system is to be fully deployed? And what are the requirements for a favorable PC status in the future?; and (4) Terminal question phase: In this phase, the participants were told that the researcher’s questions and topics were over, and if you have any further questions or tips, please express them (Polit and Beck, 2004).

*Data analysis*

Immediately after each interview, interviews were transcribed and reviewed several times to gain an understanding of the whole interview. The transcribed interviews analysis method was a five-step framework analysis (Ritchie and Spencer, 2002), which included the following steps: (1) Familiarization with the interview: This step involved immersing in the data and listening and reading the interview several times, (2) Developing a working analytical: At this stage, a thematic framework of key topics was prepared, (3) Indexing: Data structuring was done at this stage, (4) Charting: At this stage, a chart was drawn for each subject and data was transferred to it, and (5) Interpreting the data: At this stage, the relationship between the codes, subcategories and categories was explained. The extracted codes were classified according to the reduction and condensation process using MAXQDATA 10 software.

*Trustworthiness*

The findings of the qualitative content analysis should be trustworthy and any studies should be evaluated in accordance with the way the findings are generated (Polit and Beck, 2004). Credibility was established using the two methods of member checking and peer checking. For peer checking, the interviews were read several times by the research team, and after coding, the interviews were checked with the participants, and they were asked to provide corrective comments on the codes. For peer checking, data were coded and classified independently by the first author and the extracted categories were analyzed. It was then sent to the research team. In cases of disagreement, the debate between the research team continued until the consensus was reached. When the authors disagreed, discussion and clarifications continued until consensus was reached.

*Ethical consideration*

The present study is part of the first author’s Ph.D. thesis, approved by the Ethics Committee of Baqiyatallah University of Medical Sciences (BMSU) under the IR.BMSU.REC.1397.021 No. Interviews were conducted after the participants were informed about the purpose of the study and signed informed consent. Participants were also assured that data would remain confidential and anonymous.

*Findings*

Participants included 17 individuals. Table 1 shows the demographic information of the participants. A total of 457 codes were extracted from the interview analysis. The coding process was performed as a continuous comparative analysis after eliminating duplicate codes and merging similar codes based on the public health framework (Table 2).

*Policy-making*

According to the participants’ statements, current policy-making challenges included four subcategory as bellow:

*Weakness in the stewardship and centralized sovereignty of the health system*

According to the experiences of the participants, the MOHME as the health system Steward has not been able to successfully deliver PC services at community and hospital level. For example, one participant said, “Unfortunately, in recent years, hospitals and universities are acting as if they have authority of people society. (Participant 7, center manager and service provider).

*Weakness in updating the health system in accordance with the diseases patterns*

In addition to the weaknesses in stewardship and sovereignty, the weakness in updating the health system in accordance with the changes in diseases patterns was another subcategory. This reflects the weaknesses in the adaptive health system policies with the challenges ahead. “... As you know, we are facing an epidemic of aging in the near future, so we have to see what did the MOHME do to solve such a problem, ... In my opinion, the MOHME has been performed poorly so far, it is true that the authorities are talking about the importance of the phenomenon of aging and NCDs in the media, but it is important to see what they did in practice and what are their plans for the future... “(Participant 4, researcher and faculty member).

*Weakness in the coordination of decision-making bodies*

Many experts have identified the cooperation and coordination of decision-making bodies as an important factor in the success of creating and enforcing PC policies. In this regard, one participant stated, “... One of the problems of the Ministry of Health is that different deputies do not talk to each other, that is, the deputy of treatment is doing something that the social deputy does, and the deputy of nursing does something else ...” (Participant 12, center manager and service provider).

*Intensification and emergence of new international sanctions*

US sanctions against the Islamic Republic of Iran in 2018 have created serious challenges for the entire health system and subsequent PC. “... Well, international sanctions are still contributing to such a situation and may overshadow accessing to equipment’s and facilities and even human resources education...” (Participant 9, researcher and faculty member).

*Program Implementation*

Participants’ experiences indicated weaknesses in implementing PC programs and, in this respect, four subcategories were extracted.

*Failure to manage infrastructure*

Participants believed that lack of adequate infrastructure such as trained human resources, financial resources, proper referral system, and proper enforcement policies created serious problems in this filed. One of the participants stated that “... lack of infrastructure in the sense that I’m saying: lack of educational and specialized infrastructure and a coherent structure in which care is provided such as budget allocation, appropriate policy-making, etc.” (Participant 8, center manager, and service provider).

*Failure to implement PC in the form of new set ups*

Establishing PC delivery structures in the form of home care, outpatient clinics, hospice, and inpatient wards to provide these services to patients with life-threatening diseases is considered as one of the future needs of the health system of Iran, such that providing affordable and community-based services to people in collaboration with medical and nursing groups is essential. In this regard, one participant stated: “... of course some actions have been performed in the Ministry of Health ... but the hospice form, outpatient clinics, and even home care are not currently legally defined in Iran. We think it might be cost benefit in the system of Iran ... however that is a huge gap...” (Participant 2, center manager and service provider).

*Weaknesses in providing comprehensive health care*

According to the participants’ experiences, the health system of Iran does not cover the services of all stages of life from birth to death. Providing services from birth to life requires services delivery in the form of “comprehensive public health services” with the priority of health and prevention over treatment and based on the PHC. In this regard, one participant stated, “... we need to provide comprehensive services to patients at all stages of life, especially at the end of life, to define and gradually improve theses services ...” (Participant 11, policy-maker and decision-maker).

*Weaknesses in integrating PC into PHC*

In order to provide an effective PC, it is essential that these services be provided at the first level of service delivery in the health system, i.e. PHC. These patients should be identified through the Family Physician Program of comprehensive health centers and be referred to home care units, clinics, or hospitals through an appropriate service classification and referral system. One of the participants stated: “…Unfortunately, many patients from cities and villages refer to large hospitals in Tehran. We need to send these patients home and, if needed, refer them to comprehensive health centers or the nearest hospital where they live through the family physician system ... “(Participant 11, policy-maker and decision-maker).

*Comprehensive education*

 Education is an important part of health services. Thus, the three subcategories of weaknesses in integrating PC education into health education, weaknesses in providing educational resources, and weaknesses in awareness were included in this category.

*Weaknesses in integrating PC education into health education*

Many experts emphasized the importance of integrating PC education into various medical sciences, such as medicine and nursing. They explicitly stated that current education is not responsive to the present and future health status of the community and that somehow medical and nursing education should lead to community-based and home-based services delivery. For example, one participant said: “... I think it is more important than ever for PC to enter the education system for nursing and medical students ... and also a master’s degree should be defined through which PC can be teach to students from different disciplines... “(Participant 15, policy-maker and decision- maker).

*Need for providing educational resources*

Experts acknowledged that it is necessary to prepare required educational resources such as curriculum, service packs, and guidelines to integrate PC education effectively into medical education. “... In fact much of what we have now goes back to the efforts of PhD students and their research team (i.e. service packages) to develop different systems; however, a series of guidelines has also been localized in this sense ...” (Participant 9, researcher and faculty member).

*Weakness in awareness (public, stakeholders and policy-makers)*

According to the participants’ experiences, in order for an effective implementation of PC in the country, it is necessary to start with educating the professionals and policy-makers of the health sector, and then educate other stakeholders such as physicians, nurses, the health team as well as the community and families. Service providers must be aware of the challenges and necessities facing the health system, such as providing affordable services in the form of comprehensive public health services with the priority of health and prevention over treatment and based on PHC “. ... Because culturalization needs to be done by professionals. Then it comes to the community little by little. There was no congress that we didn’t attend. We just went and talked to different professionals, whether the specialists in the super-specialized fields of medicine or psychology. We attended every seminar, conference, and meeting that we could. Sometimes, we explained these individually and face to face... “(Participant 3, center manager and service provider).

*Drug availability*

According to the participants’ statements, drug availability is one of the most important components affecting the effective PC delivery. Two serious drug availability challenges were identified.

*Weakness in drug use regulations in the centers providing PC*

In order to establish PC in the health system of Iran, it is necessary to develop regulations and guidelines for the administration of narcotic and sedative drugs at home, at LTC centers and in Hospices. There is almost no planning in this regard. “... One of the serious problems we have with prescribing drugs is ,for example, when a nurse comes patient’s home for a visit, he/she really doesn’t know whether to inject morphine or not for the patients with pain. If the patient is faced with a problem, such as blood pressure drop, the law will support the nurse or not ...” (Participant 5, center manager and service provider).

*Inappropriate use and prescription by drug providers*

According to the participants’ viewpoints, physicians and nurses had a poor knowledge of drug prescriptions. “... also, the major problem in Iran is the lack of access to opioids, on the one hand, and the negative attitude of care providers, including physicians and nurses, on the other hand. A wide range of these drugs are not available to consumers. These have led to many challenges in PC in our country again ... “(Participant 10, policy-maker and decision-maker).

## Discussion

The establishment of the PC system is one of the goals of the health system of Iran. Existing studies have mentioned the necessity to modify and extend PC at home, specialized PC, modification of rehabilitation services, insurance system definition, appropriate referral system, poor teamwork quality, and lack of PC training guidelines as the PC challenges in the health system of Iran (Khoshnazar et al., 2016, Ansari et al., 2019). In this study the present status and the future challenges were extracted based on the WHO Public Health Strategy model into four categories of policy-making, implementation, comprehensive education, and drug availability.

The first extracted category was PC policy-making. Ansari et al. argued that PC stewardship should provide appropriate mechanisms for cross-sectional cooperation for the participation of organizations outside the MOHME, insurer organizations, legal entities, legislation (Ansari et al., 2019). The second subcategory was the weakness in updating the health system in accordance with the changes in diseases patterns. Although appropriate changes have been introduced and implemented such as the Family Physician Plan in 2005 (Salavati et al., 2018), the creation of the PC working group in 2013 (Ansari et al., 2018), the Health System Evolution Plan in 2015 (Salavati et al., 2018), many disadvantages and challenges such as insurance coverage of services, comprehensive services, appropriate policy-making, and financing of these projects has led to lack of full implementation of these plans and, despite their beneficial effects, presents serious challenges to the public health system. The other subcategory was the lack of coordination of decision-making bodies. The results of the present study showed that different deputies of the MOHME individually developed working groups and activities in PC, in which there is no roadmap. In this regard, Herman et al., (2019) considered the need for more serious interactions between organizations and the creation of communication channels and leadership development as a major challenge for PC delivery. The intensification and emergence of new international sanctions was the last PC policy-making subcategory. In this regard, Aloosh et al., study (2019) showed that economic sanctions have negative effects on the health of Iranian society by damaging social determinants of health and access to drugs and care. These sanctions have caused economic inequality and health gaps. Therefore, it seems that PC policy-making in the health system of Iran is in its early stages and the attention of the authorities and policy-makers need to be focused on overcoming the stated challenges in the future.

The next category, namely PC implementation, involved four serious challenges. Considering the subcategory of failure to manage developed infrastructure, WHO has introduced six axis related to the design and development of PC system infrastructure in 2016. These six axis include policy-making, funding, service delivery, human resource development, drug availability, and information and research (WHO, 2018). Regarding the subcategory of failure to implement PC in the form of new set ups, Khoshnazar et al., (2016) have stated that the health system of Iran requires home PC, specialized PC, modification of rehabilitation services, insurance system definition, and appropriate referral system. In 2012, the MOHME has formulated a comprehensive national program of supportive and PC for cancer in line with the two above-mentioned subcategories, but so far (2019), PC delivery infrastructure has not yet been well established, indicating weakness in the management structure. With regards to the subcategory of weakness in providing comprehensive health services, a comprehensive and inclusiveness services were placed on the agenda of the MOHME of Iran in 2015 during the Health System Evolution Plan (Rassouli and Sajjadi, 2016a). These services should be provided in the form of service packages for different age groups and in different health conditions. Despite efforts made by various deputies of the MOHME, such as deputy of treatment and deputy of nursing, the weaknesses in the formulation as well as the tariff of service packages for end-stage diseases, mostly of the elderly population, constitute a serious challenge for the Iranian health system and society (Jabbari et al., 2019). It is anticipated that it will face much more serious challenges in the near future. In the case of the subcategory of weakness in integrating PC into PHC, the plan to integrate the family physician into the health system of Iran has been carried out in 2005. Jabbari et al., (2019) stated that most family physicians in Iran, do not have sufficient involvement to provide PC to patients. Therefore, it seems that the future challenges of PC are in line with the challenges of the health system of Iran such that these services can be provided as a comprehensive, complete, and inexpensive service to patients in line with the family physician plan.

Comprehensive PC education was the third category extracted. In this regard, Callaway et al., (2018) have noted that PC education subgroups should include medical, nursing, psychology, social worker etc. and responsible for reviewing the undergraduate and graduate curricula. Curricula should also be translated into local languages and be tailored to the culture of each community. For example, Tajikistan, with the support of the Tajik School of Nursing and Medicine, translated the program of the American Association of Colleges of Nursing’s “End-of-Life Nursing Education Consortium” into Russian. Education should include all health system managers, government officials, legal experts, pharmacists, the media, spiritual leaders, human rights defenders, the police and the general public. According to the participants’ statements, PC education is another serious challenge for PCs such that the structure is not yet well positioned in the medical education system, and nurses, physicians and other related disciplines have not received effective training in this field. PC curriculums, training packages, and guidelines are also in the elementary stage. On the other hand, stakeholder, patients’ family, policy-makers, and PC providers have a more favorable status in these trainings than in the past, but there is still a long way towards the ideal situation.

The last extracted category was drug availability. According to the International Narcotics Control Board (INCB), Iran ranks 115^th^ in the world and 25^th^ in Asia and is one of the least consumed countries (Rassouli and Sajjadi, 2016b). Cleary et al., (2013) have noted that most countries in the Middle East have very restrictive laws on access to opiates. There are some challenges for drug availability including inadequate education and poor training of medical professionals, limited funding, and cultural challenges in many countries (Rassouli and Sajjadi, 2016a, Silbermann, 2014, Silbermann et al., 2015) The study by Rassouli and Sajjadi (2016b) also noted that poor access to opioid drugs as well as the negative attitudes of physicians, patients, and their families to these drugs is a serious challenge. Therefore, like other countries, Iran seems to have the challenge of limited access to narcotic drugs, poor knowledge of service providers as well as the adoption of rules on the use of these drugs. It requires an all-out effort to put in place appropriate legislation and policy-making for the use of these drugs.

In conclusion, Although, this study sought to provide a rational view of the present status and the future challenges of PC based on the views of managers and policy-makers of the health system of Iran, there are still many unknown issues about the performance of PC system as well as the slow development of this system in response to increased elderly population and the needs of patients with disabilities. Due to an increase in the elderly population and increase in NCDs, Iran will soon face the challenge of access to end-of-life care and PC for patients with life-threatening disease. Therefore, it is suggested that authorities focus on the challenges of integrating PC into the health system and manage these challenges by using comprehensive and foresight methods.

**Table 1:** Demographic Information of Participants

Age (Mean ± SD؛ years)	48.35±9.98
PC activity history Mean ± SD؛ years	6.23±2.1
Average interview time (Mean ± SD ؛ minutes)	33.35±7.22
Number of interviews (Mean ± SD)	17
Sex	
Male	7(1.2%)
Female	10(58.8%)
Education	
B.A.	5(29.4%)
M.A.	3(17.6%)
Ph.D.	9(52.9%)
Type of related activity	
Policy-maker and decision-maker	8(47.1%)
Faculty member and researcher	4(23.5%)
Center manager and PC provider	5(29.4%)

**Table 2:** PC Challenges in Iran

Main category	Subcategory
Policy-making	Weakness in the stewardship and centralized sovereignty of the health system
	Weakness in updating the health system in accordance with the diseases patterns
	Weakness in the coordination of decision-making bodies
	Intensification and emergence of new international sanctions
Program implementation	Failure to manage infrastructure
	Failure to implement PC in the form of new set ups
	Weaknesses in providing comprehensive health care
	Weaknesses in integrating PC into primary health care(PHC)
Education	Weaknesses in integrating PC education into health education
	Weaknesses in providing educational resources
	Weaknesses in awareness (public, stakeholders and policy-makers)
Drug Availability	Inadequacy / inefficiency of drug use regulations in the centers providing PC
	Inappropriate use and prescription by drug providers
